# Insights into Inhibitory Effects of Lapachol against Zika Virus

**DOI:** 10.1021/acsomega.6c04563

**Published:** 2026-10-05

**Authors:** Stephanie Paola Borjas Reyes, Natasha Marques Cassani, João Victor Rodrigues Dutra, Maria Eduarda Trevisan Aguillar da Silva, Nathalya Cristina de Moraes Roso Mesquita, Caroline Martins Mota, Tiago Wilson Patriarca Mineo, Glaucius Oliva, Rafael Victorio Carvalho Guido, Bruno Amaral Meireles, Diego Pandeló José, Ana Carolina Gomes Jardim

**Affiliations:** † Laboratory of Antiviral Research (LAPAV), Institute of Biomedical Science (ICBIM), Federal University of Uberlândia (UFU), Uberlândia, MG 38405-302, Brazil; ‡ 28114 Federal University of Minas Gerais, Institute of Biological Sciences, Department of Genetics, Ecology and Evolution, Belo Horizonte, Minas Gerais 31270-901, Brazil; § Federal University of Triângulo Mineiro, Iturama, Minas Gerais 38280-000, Brazil; ∥ São Carlos Institute of Physics, University of São Paulo, São Paulo 13563-120, Brazil; ⊥ Laboratory Immunoparasitology ″Dr. Mário Endsfeldz”, Federal University of Uberlândia (UFU), Uberlândia, MG 38405-302, Brazil

## Abstract

The reemergence of Zika virus (ZIKV) in 2015 and its association with severe neurological manifestations highlighted the urgent need for effective antiviral therapies, which, despite extensive efforts, remain unavailable. Lapachol, a 1,4-naphthoquinone compound abundant in Brazilian plant species, seems to be a promising candidate as an antiviral agent since its activity against other RNA viruses, as well as other human pathogens, including reported activity against Epstein–Barr virus and inhibition of herpes simplex virus type 1 by a lapachol-derived compound. Here, we investigated the activity of lapachol in inhibiting ZIKV replication by employing the wild-type strain ZIKV_PE243_. Lapachol exhibited potent inhibition of ZIKV with an EC_50_ of 3 μM and selectivity index of 50. Mechanistic assays suggest that its main action is exerted through postentry inhibition of viral replication, possibly through the blockage of the catalytic sites of NS2B-NS3 protease and NS5 polymerase. Biochemical enzymatic assays confirmed that lapachol inhibits NS2B-NS3 protease and NS5 polymerase activities, being complemented by molecular docking analyses. A protective effect was noted when cells were treated prior to infection, and lapachol was found to induce ROS formation *in vitro*, indicating that several antiviral mechanisms may be involved, including the initiation of an oxidative stress response that can affect viral particles. Overall, these findings shed light on the potential of lapachol to be used for the development of ZIKV inhibitors, especially targeting postentry stages of virus replication.

## Graphical abstract

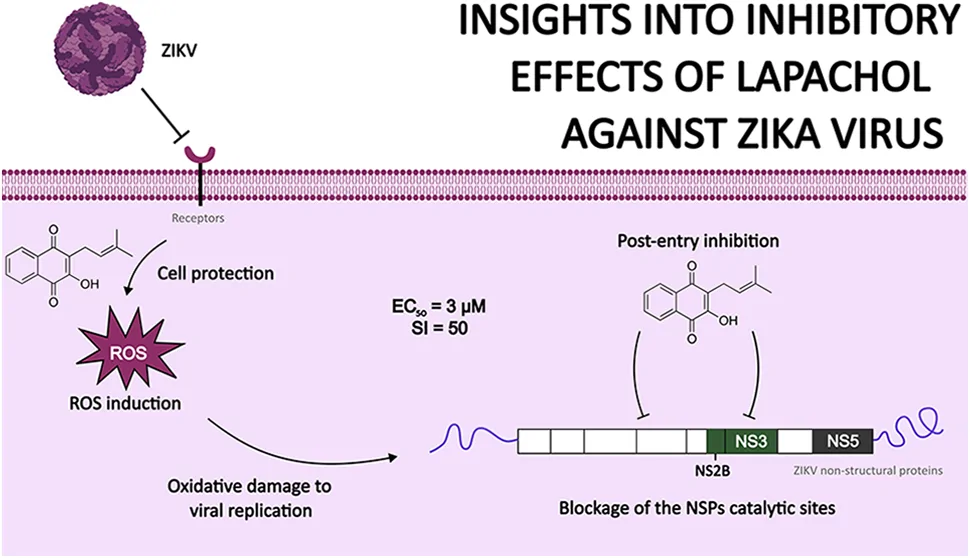

## Introduction

Among infectious diseases that cause significant health challenges in tropical countries, Zika fever stands out for its ability to cause long-lasting consequences in affected individuals.^1,2^ Caused by *Orthoflavivirus zikaense* (Zika virus, ZIKV, *Flaviviridae* family), it is an asymptomatic disease in 80% of cases, and when symptomatic, mild symptoms like low-grade fever, joint pain, headache, asthenia, and maculopapular rash are mainly observed, typically following a self-limiting course.^2,3^

ZIKV is a single-stranded positive-sense RNA arbovirus that belongs to the family *Flaviviridae*, transmitted to humans through the bite of the mosquito *Aedes aegypti*. ZIKV is also transmitted through other documented routes, including sexual contact, vertical (mother-to-child) transmission, blood transfusion, and potentially breast milk.^4,5^ The vertical and sexual routes are of particular public health relevance, as they account for cases in nonendemic regions and for the severe neurological consequences observed in neonates.^6^ ZIKV genome has one open reading frame (ORF) that codifies for a single polyprotein, that is later proteolytically cleaved to produce three structural proteins, the envelope protein (E), the capsid protein (C), and the membrane protein (M), as well as seven nonstructural proteins that are essential for virus replication into the host cells.^7^ Because of their important role in infection establishment, nonstructural proteins, with essential enzymatic function for virus replication, are being widely studied as targets for antiviral development.^8^ In this sense, NS3 stands out since its N-terminal domain (NS3^Pro^), together with NS2B, forms a trypsin-like serine protease that is responsible for cleaving the viral polyprotein. Its C-terminal domain possesses helicase and NTPase activities that help unwind secondary RNA structures on the 5′-UTR, a function necessary for virus initial replication.^7^ Another potential target is the NS5^RdRp^, a ZIKV RNA-dependent RNA polymerase (RdRp), that functions as a key component of the virus replication complex and plays several critical roles in the viral life cycle. It is also a multifunctional protein with two main domains: a N-terminal methyltransferase (MTase) domain responsible for mRNA capping and a C-terminal RNA-dependent domain responsible for viral RNA synthesis. In other viruses, inhibition of NS5 has been achieved by nucleoside analogs and polymerase inhibitors.^9,10^

In this sense, compounds extracted from natural sources have become of increasing interest, with many of them showing antiviral activity against viral proteins.^11–14^ Quinones are a well-studied group of compounds that are known for their diverse physicochemical and bioactive properties. They can be classified based on the amount of benzene rings found in their structure: benzoquinones (contain one ring), naphthoquinones (contain two rings), or anthraquinones (contain three rings). Naphthoquinones, produced as secondary metabolites by a wide range of species among different kingdoms,^15^ contain a naphthalene ring (fusion of a pair of benzene rings) with two carbonyl groups in the 1,4-positions.^16^ Lapachol is a 1,4-naphthoquinone that can be found in many plant species native to South America, with greater abundance in trees of the genus *Roseodendron, Tabebuia*, and *Handroanthus*, such as *Handroanthus impetiginous*. For its wide distribution and bioactive properties, lapachol has been extensively studied, demonstrating its versatile role as an antibacterial,^17^ antifungal,^18^ and antitumoral^19^ agent, with scarce reports of its antiviral activity.^20,21^ In this sense, the purpose of our study was to evaluate the effects of lapachol on ZIKV replication *in vitro* with insights into its mechanism of antiviral action.

## Results and Discussion

### Lapachol Exhibits Potent Efficacy and Low Cytotoxicity

Aiming to explore the eligibility of lapachol as an antiviral candidate against ZIKV, we evaluated its cytotoxicity profile and antiviral activity using Vero E6 cells in the presence or absence of infection with ZIKV_PE243_ performing a 2-fold serial dilution. Results revealed that a low compound concentration was sufficient to achieve 50% of its maximum effect as indicated by an EC_50_ of 3 μM. In contrast, a high concentration was required to induce toxicity in 50% of the cell population, showed by a CC_50_ of 150.1 μM, resulting in a Selectivity Index (SI) of 50 (Figure 1). These results suggest that lapachol acts as a strong inhibitor at low concentrations with high selectivity.

**Figure 1:**
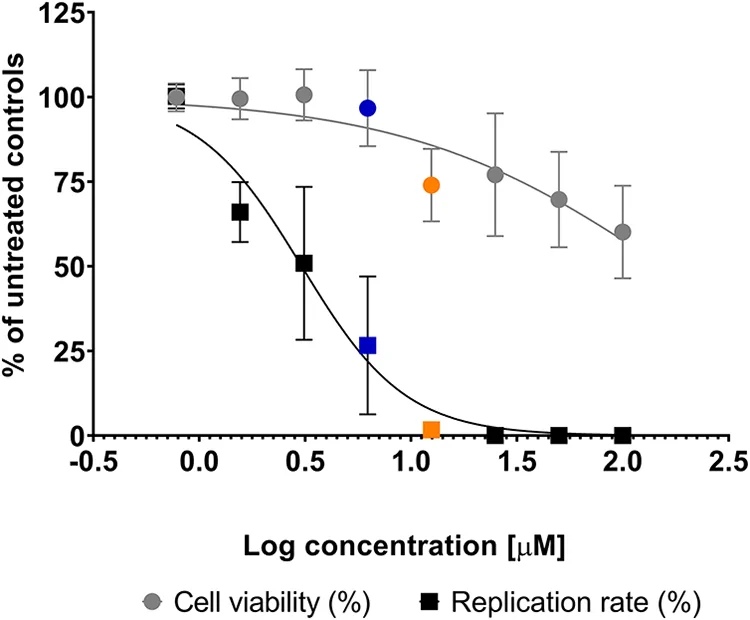
Dose-dependent effect of lapachol on ZIKV replication. Vero E6 cells were treated with 2-fold serial dilutions of lapachol ranging from 0.78–100 μM in the presence or absence of ZIKV_PE243_ infection at an MOI of 0.01. The CC_50_ was measured by MTT assays (gray circles) and EC_50_ was calculated by quantification of FFU (black squares). The concentrations of 6.25 and 12.5 μM are represented in blue and yellow, respectively. Mean values of three independent experiments each measured in triplicate including the standard deviation are shown. CC_50_ and EC_50_ values were calculated by nonlinear regression using GraphPad Prism 8.0.

Based on these, a working noncytotoxic concentration that inhibited over 80% of virus replication was selected for further assays. As observed in Figure 1, this concentration was found between 6.25 μM (inhibition rate = 74%; cell viability = 97%), represented in blue, and 12.5 μM (inhibition rate = 98%; cell viability = 74%), represented in yellow. Therefore, considering the dose–response profile of the compound, the concentration of 8 μM was selected for further experiments.

### Lapachol Confers Protection to Vero E6 Cells against ZIKV Infection

As an approach to elucidate the potential targets of lapachol, we exposed the virus to the compound at 8 μM at different time points of the infection. An initial evaluation of the host-directed activity of lapachol was performed through pretreatment assays, where cells were first incubated with the compound for 2 h at 37 °C prior infection (Figure 2). Results revealed that lapachol can confer significant protection to the cells with a 100-fold reduction of the supernatant viral titer (*p* < 0.01) (Figure 3A). These results suggest that one mechanism of antiviral action of lapachol may be related to the interaction of host elements hindering ZIKV infection. It is worth noting that the dose–response antiviral assay was performed at a low MOI (0.01) to capture cumulative, multicycle inhibition, whereas the time-of-addition mechanistic assays used higher MOIs (1 and 3) to ensure reliable infection during the short 2 h adsorption windows.

**Figure 2:**
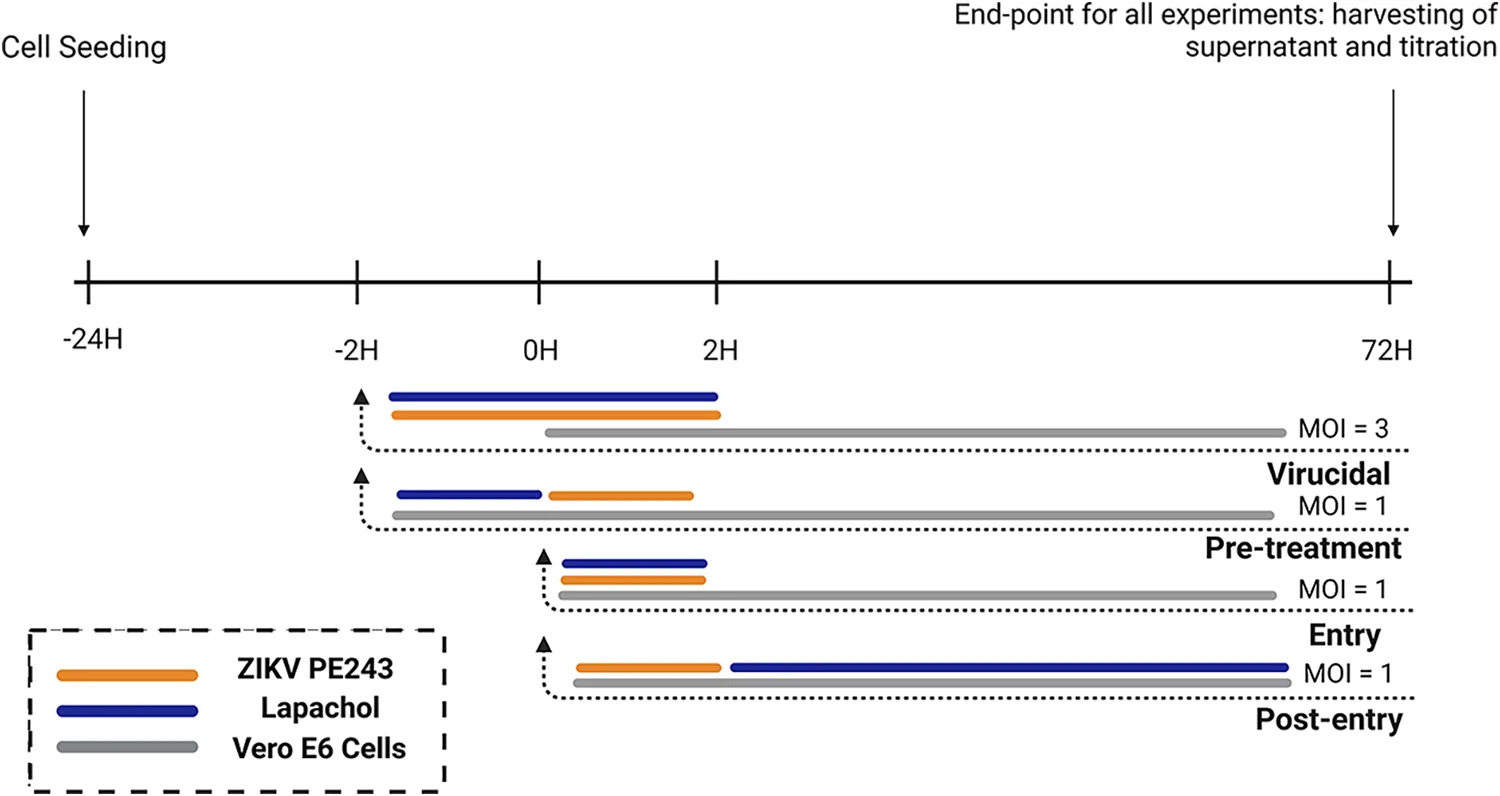
Schematic representation of time-of-addition assay. Vero E6 cells (gray lines) were treated with 8 μM of lapachol (blue lines) at different time points of ZIKV (yellow lines) infection. All assays were followed by supernatant collection and titration 72 h.p.i.

**Figure 3:**
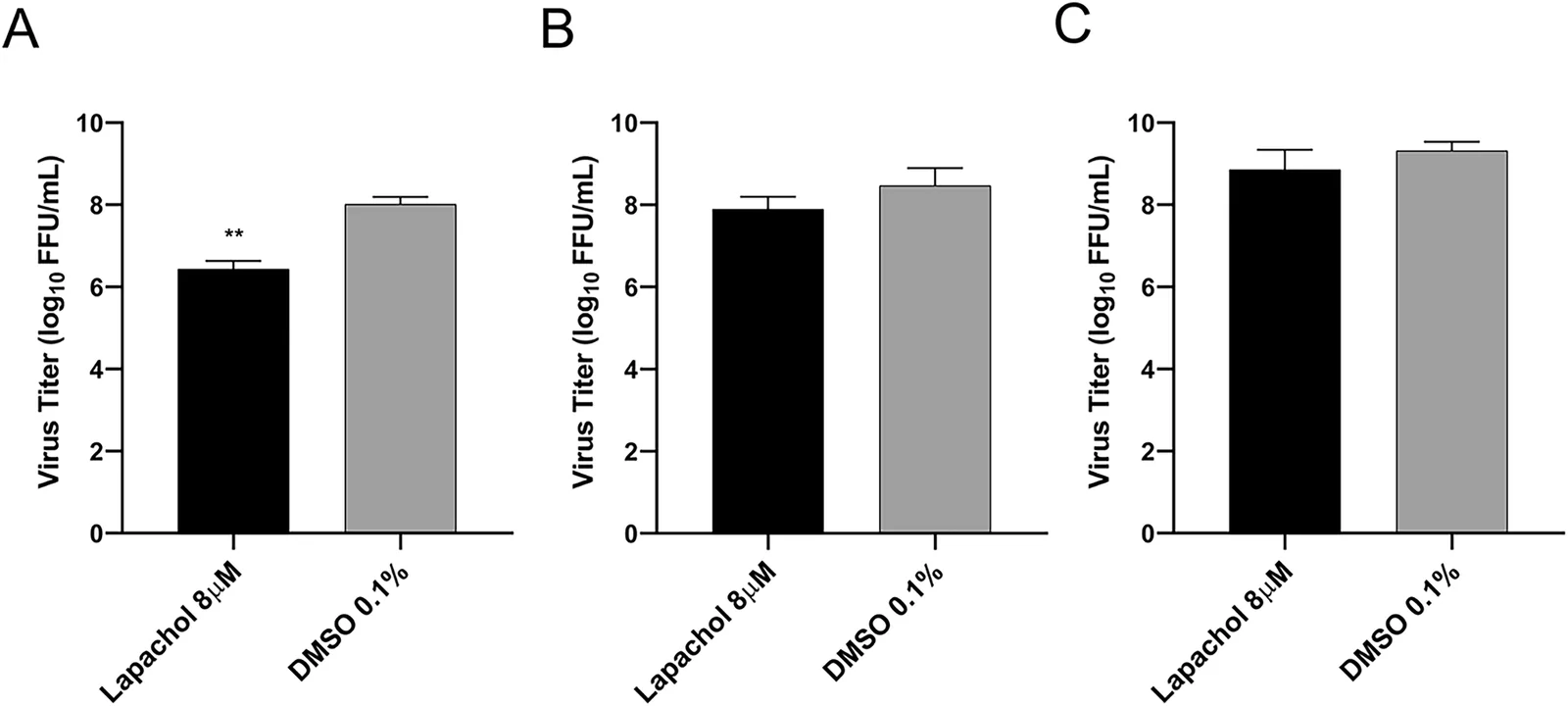
Lapachol confers protection to Vero E6 cells against ZIKV infection. (A) Vero E6 cells were treated with the compound at 8 μM for 2 h at 37 °C, followed by compound removal and ZIKV_PE243_ infection (MOI = 1) for 2 additional hours. Cells were washed with PBS [1×], and virus-free medium was added. (B) Virucidal effect was assessed by exposing ZIKV_PE243_ (MOI = 3) to the compound at 8 μM for 2 h at 37 °C with subsequent infection of Vero E6 cells with this inoculum for extra 2 h. (C) Entry assays were performed by adding lapachol and ZIKV_PE243_ simultaneously and incubating for 2 h at 37 °C. Schematic representation on the top of graphics as follows: Vero E6 cells (gray lines), lapachol (blue lines), ZIKV (yellow lines). Data are expressed as virus titer in logarithmic form. Mean values of three independent experiments each measured in triplicate including the standard deviation are shown. DMSO 0.1% was used as untreated control. *P* values <0.05 were considered significant. (*) *p* < 0.05, (**) *p* < 0.01, (***) *p* < 0.001, and (****) *p* < 0.0001. Images were generated with BioRender.com and GraphPad Prism 8.0.

Early stages of the ZIKV infection were also evaluated. The effect of lapachol over ZIKV particles was assessed by incubating ZIKV with the compound for 2 h, proceeding to the infection of the cells for an additional 2 h. Alternatively, to investigate virus entry to host cells, ZIKV and lapachol were simultaneously added to cells for 2 h. For both protocols, the inoculum was then removed, cells washed, and incubated with fresh media (Figure 2). No virucidal effect was observed (Figure 3B). Additionally, entry/attachment assays revealed that the compound is not able to impair virus entry to the cell (Figure 3C). It should be noted that, in this coincubation design, lapachol and ZIKV are added together to the cells, so the assay assesses viral attachment and entry collectively and does not discriminate between inhibition of surface attachment and of postattachment entry steps. The absence of significant inhibition therefore indicates that lapachol does not interfere with either the early attachment or the subsequent entry of ZIKV at the tested concentrations.

### Lapachol Strongly Impairs ZIKV Replication after Virus Entry to Host Cells

As an evaluation of the interference of lapachol on late stages of ZIKV replication cycle, postentry assays were performed by infecting cells for 2 h, washing them to remove unbound virus and then treating the cells with the compound at 8 μM (Figure 2). As seen in Figure 4, lapachol was able to effectively and strongly inhibit ZIKV replication after virus entry to the cell, leading to undetectable levels on its supernatant, in comparison to a mean viral titer of 1 × 10^9^ FFU/mL in untreated cells (*p* < 0.01). Therefore, lapachol was able to reduce ZIKV replication in 9 log_10_, when added to the cells after virus entry (Figure 4).

**Figure 4:**
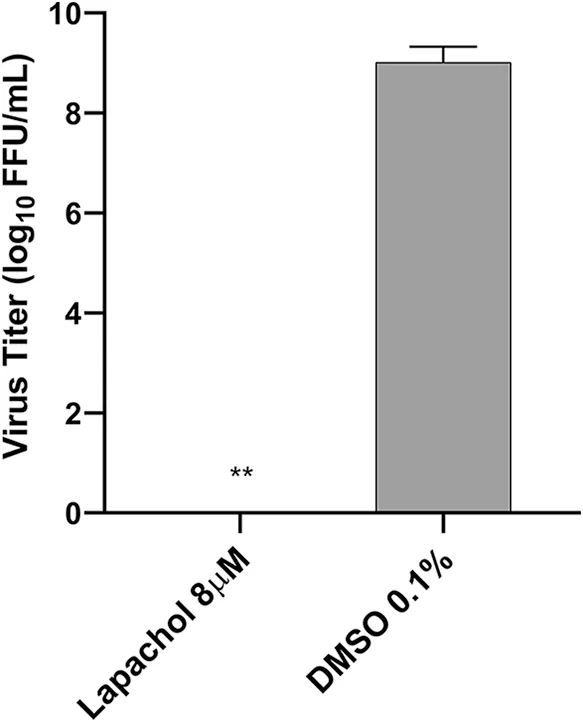
Lapachol strongly inhibits postentry stages of ZIKV replication in Vero E6 cells. ZIKV_PE243_ at a MOI of 1 was added to cells for 2 h at 37 °C, cells were washed with PBS [1×], and the inoculum was replaced by medium containing lapachol at 8 μM. Supernatant was titrated 72 h.p.i. Schematic representation on the top of graphics as follows: Vero E6 cells (gray lines), lapachol (blue lines), ZIKV (yellow lines). Data are presented as virus titer expressed in logarithmic form. The mean values of three independent experiments each measured in triplicate including the standard deviation were compared. DMSO 0.1% was used as untreated control. *P* values <0.05 were considered significant. (*) *p* < 0.05, (**) *p* < 0.01, (***) *p* < 0.001, and (****) *p* < 0.0001. Images generated with GraphPad Prism 8.0.

### Lapachol Interferes with NS2B-NS3^Pro^ and NS5^RdRp^ Enzymatic Activity

ZIKV NS3^Hel^, NS2B-NS3^Pro^, and NS5^RdRp^ were initially submitted to end point activity assays; lapachol was initially tested at 1 mM for NS3Hel, and at 500 μM for both NS2B-NS3^Pro^ and NS5^RdRp^. As shown in Figure 5A, lapachol exhibited a low ATPase inhibitory effect on NS3^Hel^, with less than 20% inhibition of its activity, while it inhibited 87% of NS2B-NS3^Pro^ and 91% of NS5^RdRp^ activities. To assess the concentration–response inhibitory effect of lapachol on NS2B-NS3^Pro^, concentrations ranging from 0.98 to 500 μM were tested and an IC_50_ value of 144 ± 12 μM was obtained (Figure 5B). Collectively, these findings indicate that lapachol may act by directly affecting viral protease and polymerase activities. This is consistent with previous data showing lapachol’s impact on postentry stages, as interference with NS2B-NS3^Pro^ and NS5^RdRp^ would affect virus replication.

**Figure 5:**
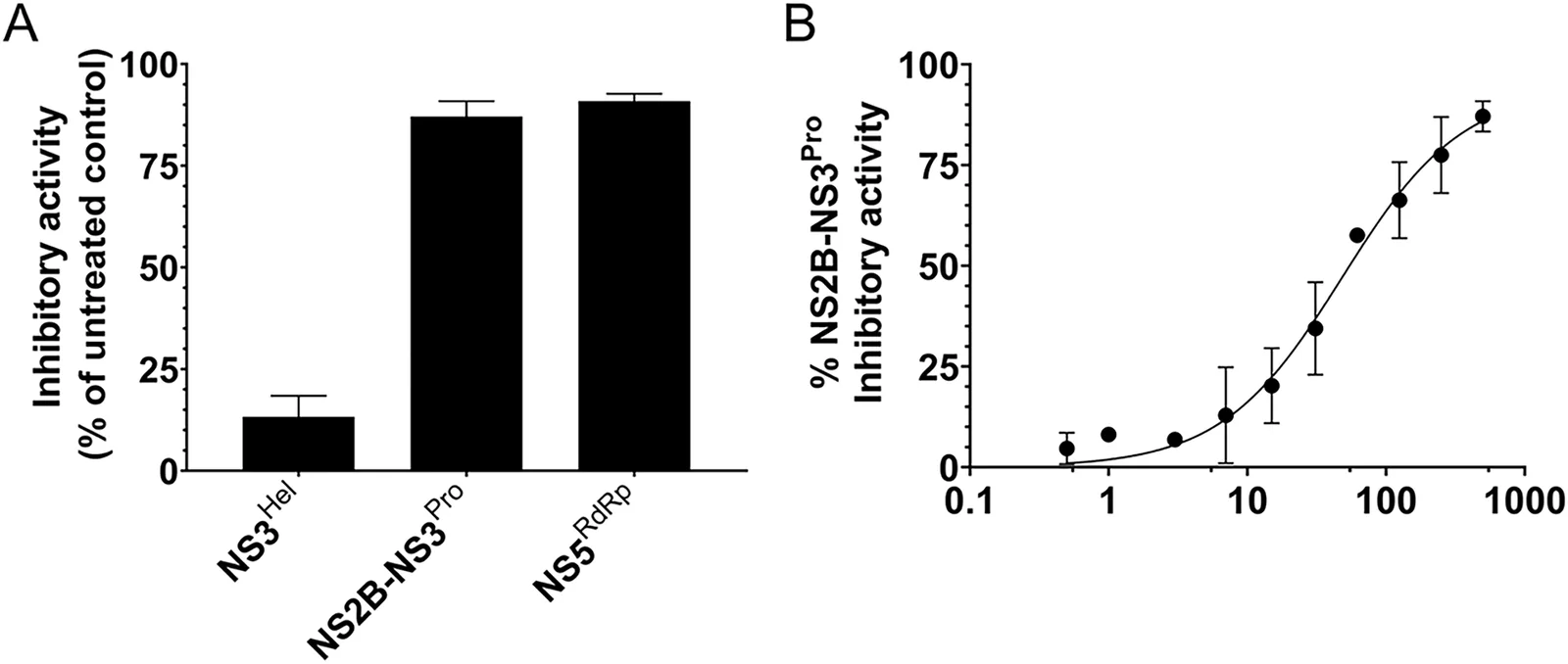
Lapachol interferes with viral nonstructural proteins essential for virus replication. (A) NS3hel: unique-point measurement of NTPase activity of 200 nmol L^−1^ ZIKV NS3Hel in the presence of lapachol at 1 mM using 1 mM of ATP as substrate. Absorbance was measured at the end of a 30 min reaction using λ = 620 nm. NS2B-NS3^Pro^: unique-point measurement of protease activity of 4 nM of purified ZIKV NS2B-NS3^Pro^ using Bz-nKRR-AMC at 30 μM as substrate in the presence of lapachol at 500 μM. Relative fluorescence was measured against a positive control reaction containing an equal concentration of DMSO. NS5^RdRp^: unique-point measurement of elongation activity of ZIKV NS5^RdRp^ by quantification of SYBR Green signal in the presence of lapachol at 500 μM. (B) Concentration–response evaluation of ZIKV NS2B-NS3^Pro^ inhibition in the presence of lapachol at concentration ranging from 0.98 μM to 500 μM. All tests were performed in biological duplicate and technical triplicate. Reactions containing DMSO 1% (v/v) were used as reference. Images generated with GraphPad Prism 8.0.

### *In Silico* Data Show That Lapachol Binds to Nonstructural Proteins Essential for Virus Replication

Molecular docking analyses suggested that lapachol is able to interact with two functionally relevant regions of the ZIKV replication machinery (Figure 6). In the NS3 protein, lapachol was predicted to bind within the protease domain, in close proximity to the catalytic triad residues His51 and Ser135. The ligand established conventional hydrogen bonds with Thr134 and Ser135, while multiple van der Waals interactions involving Asp129, Tyr130, Pro131, Gly133, Tyr150, and Val52 further contributed to ligand stabilization within the binding pocket. Additional hydrophobic and aromatic interactions included a π-sigma interaction with Ala132, π-alkyl interactions with His51 and Val36, and a π–π stacked interaction with Tyr161, reinforcing the binding pose (Figure 6A). In the NS5 protein, lapachol was predicted to occupy a pocket adjacent to the priming loop of the RNA-dependent RNA polymerase (RdRp) domain, a region essential for the initiation of viral RNA synthesis. The ligand was stabilized primarily through hydrophobic and aromatic interactions, including π–π T-shaped interactions with Trp805 and Lys802, an amide−π stacked interaction with Lys802, and π-alkyl contacts involving Arg794 and Lys802. Additionally, a π-sulfur interaction with Met806 further contributed to ligand stabilization within the binding pocket. The binding pose was also supported by multiple van der Waals contacts with Ile799, Gly801, Gly803, Thr795, and Thr796 (Figure 6B). Collectively, these results indicate that lapachol may interfere with key enzymatic functions of both NS3 and NS5, highlighting its potential as a multitarget inhibitor against ZIKV replication.

**Figure 6:**
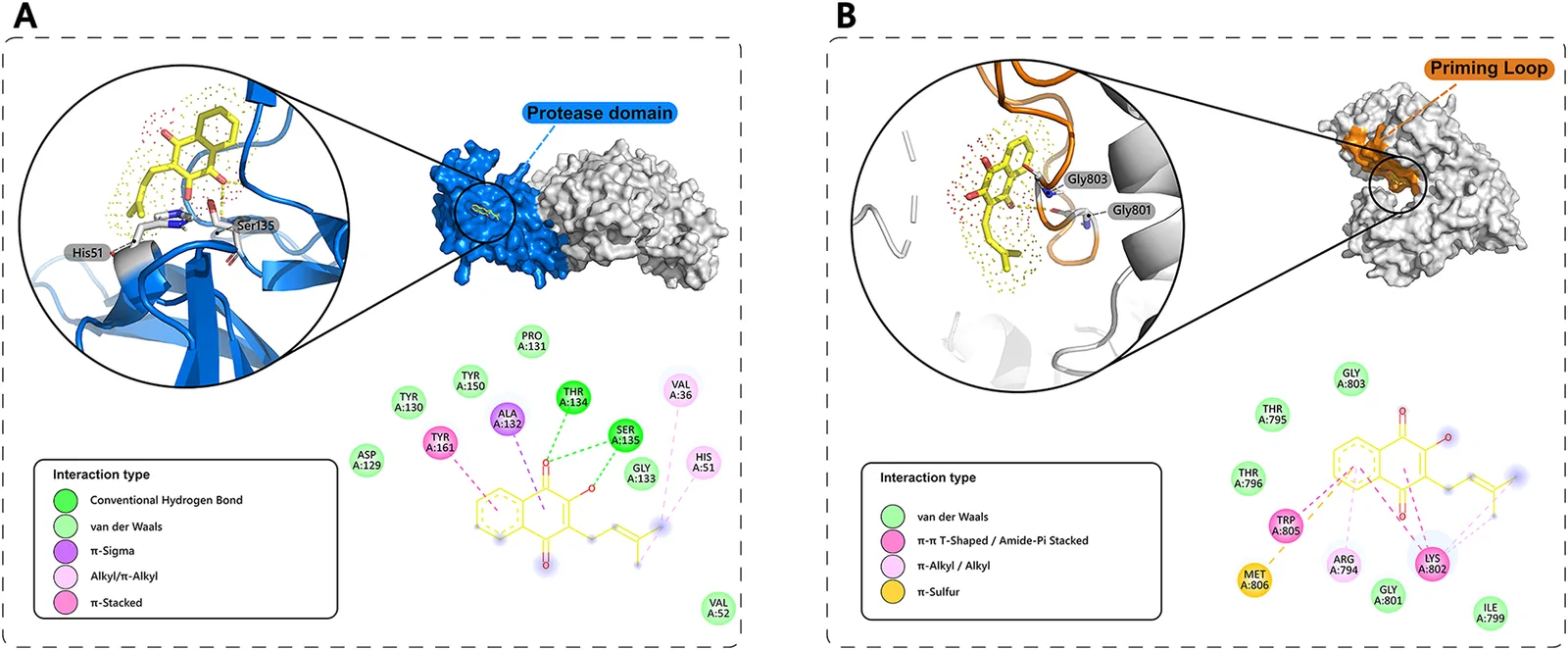
Predicted binding modes of lapachol with ZIKV NS3 protease and NS5 RNA-dependent RNA polymerase. (A) Three-dimensional representation of the docking pose of lapachol within the protease domain of the Zika virus (ZIKV) NS3 protein. The protein surface is shown with the protease domain highlighted in blue. The enlarged view shows lapachol (yellow sticks) positioned near the catalytic triad residues His51 and Ser135. The corresponding two-dimensional interaction diagram illustrates the network of conventional hydrogen bonds, van der Waals contacts, π–cation, π–sigma, and π–alkyl interactions contributing to ligand stabilization within the active site. (B) Predicted binding mode of lapachol in the ZIKV NS5 RNA-dependent RNA polymerase (RdRp). The priming loop is highlighted in orange on the protein surface, and the enlarged view depicts the ligand accommodated in a pocket adjacent to this functional region. The two-dimensional interaction map reveals van der Waals, π–π T-shaped, amide−π stacked, π–alkyl, and π–sulfur interactions involving residues located within or surrounding the priming loop region. Interaction types are color-coded according to the legends shown in each panel.

Lapachol exhibited a binding affinity of −6.232 kcal/mol toward ZIKV NS3^pro^, which is highly comparable to the value obtained for the reference inhibitor cn-716 (−7.498 kcal/mol) (Table 1). For the NS5^RdRp^ allosteric site, lapachol scored −5.690 kcal/mol, demonstrating a competitive thermodynamic profile when compared to that of the reference Cpd14 (−5.292 kcal/mol). To contextualize these results, the predicted binding modes and interaction networks of the reference inhibitors are presented in the Figure S4 (Supporting Information), showing that both CN-716 and Cpd14 occupy the same catalytically and functionally relevant regions targeted by lapachol. In addition, validation of the docking protocol through redocking of the crystallographic ligands is provided in Figures S5 and S6 (Supporting Information) where the close agreement between the experimental and predicted poses for NS3 and NS5, respectively, confirms the suitability of the docking setup used throughout this study. The estimated binding affinity scores and detailed interaction profiles for all alternative conformations (poses) generated during the molecular docking simulations of lapachol against the respective viral proteins are thoroughly compiled in Table S1. These benchmarking data suggest that lapachol fits effectively within both of the viral pockets.

**Table 1:** Binding Affinity Energies (kcal/mol) Estimated by Molecular Docking for Lapachol and Reference Inhibitors (Positive Controls) against the ZIKV NS2B-NS3 Protease and NS5^RdRp^ Proteins

target	compound	affinity (kcal/mol)
NS2B-NS3^pro^	lapachol	–6.232
	cn-716 (positive control)	–7.498
NS5^RdRp^	lapachol	–5.690
	Cpd14 (positive control)	–5.292

### Lapachol Induces Intracellular ROS Production

Since naphthoquinones such as lapachol are prone to act as oxidants, we evaluated the intracellular ROS production in cells treated with lapachol at 8 μM or with DMSO 0.1%, in the presence or absence of ZIKV_PE243_, and incubated for 72 h. As observed in Figure 7, ZIKV infection of the cells by itself induced an increment in ROS production. Additionally, cells treated with lapachol in the absence of infection also showed slightly higher levels of ROS, indicating that the compound alone is capable of inducing ROS formation. Interestingly, the treatment of infected cells with lapachol significantly elevated ROS production beyond those of the other three analyzed groups, suggesting that the infection by ZIKV and lapachol treatment presented a combined effect in ROS production.

**Figure 7:**
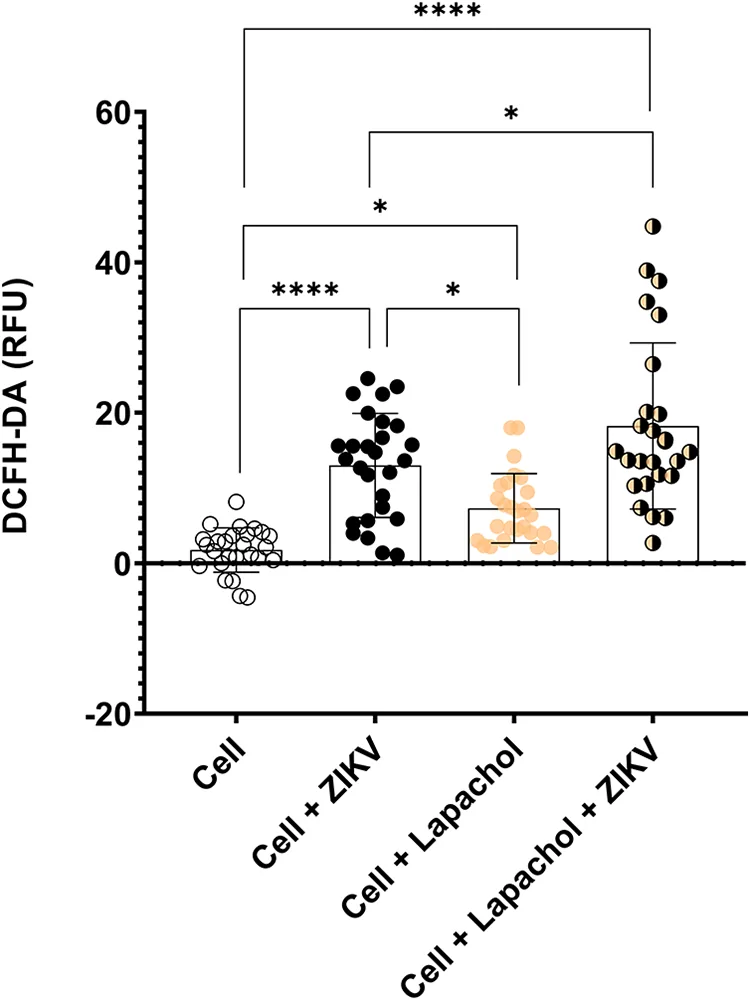
Determination of ROS production through lapachol’s treatment. Vero E6 cells were treated with lapachol at 8 μM in the presence or absence of ZIKV_PE243_ infection at an MOI of 1 and incubated for 72 h. Levels of ROS formed intracellularly were determined using a DCFH-DA (2′,7′-dichlorohydrofluorescein diacetate fluorescent probe). Infected cells are represented in black circles and treated cells are represented in orange circles. Mean values of three independent experiments each measured in replicates of nine are shown. Bars show the mean standard deviation of each group; individual values are plotted as circles. *P* values <0.05 were considered significant. (*) *p* < 0.05, (**) *p* < 0.01, (***) *p* < 0.001, and (****) *p* < 0.0001. Images were generated with GraphPad Prism 8.0.

## Discussion

This study evaluated the *in vitro* antiviral activity of lapachol against ZIKV, providing evidence of its potential mechanisms of action. The 1,4-naphthoquinone, lapachol, strongly inhibited ZIKV replication, primarily affecting postentry stages of the viral cycle, being able to reduce viral titers to undetectable levels in comparison with the DMSO control. In addition, it demonstrated protective activity by a 100-fold reduction in viral titer and protected Vero E6 cells from ZIKV infection. Evaluation of its mechanism of action suggests that it may act by inhibiting the enzymatic activity of nonstructural proteins and causing oxidative stress damage to the viral particle through ROS formation.

Naturally occurring compounds containing naphthoquinones in their structure have garnered attention from the scientific community due to their wide range of pharmacological properties as well as ample distribution in nature.^15,22,23^ Among this group of compounds, lapachol stands out for its therapeutic versatility, acting as an anticancer agent as well as an inhibitor of many pathogens.^24,25^ Due to previously reported antiviral potentials of lapachol,^20,21^ we evaluated its capacity to inhibit the ZIKV replication cycle.^20^ Lapachol exhibited substantial inhibition of ZIKV replication, being able to inhibit infection at low concentrations and presenting high tolerance by the host cells with an SI of 50.7 demonstrating the strong selectivity of lapachol against ZIKV *in vitro*. Therefore, our data correlate with previous studies where lapachol was able to interfere with Epstein–Barr virus (EBV) replication with a dose-dependent effect.^20^ It is also comparable to the antiviral activity reported for a synthetic 1,4-naphthoquinone structurally derived from lapachol, which could inhibit the replication of Herpes simplex virus type 1 (HSV-1) with an IC_50_ of 25 ng/mL and an IC_90_ of 400 ng/mL, with a therapeutic index of 2000.^28^

Further evaluation of lapachol effects on ZIKV at different stages of its replication revealed that the compound exerts a more pronounced effect after ZIKV entry into host cells. This could be attributed to the interference with nonstructural proteins of the virus, as observed when enzymatic activity of NS2B-NS3^Pro^ and NS5^RdRp^ were reduced in the presence of the compound, exhibiting an IC_50_ of 144 μM over NS2B-NS3^Pro^. Molecular docking analysis demonstrated that lapachol’s binding to NS3 is energetically favorable, primarily due to the formation of both hydrophobic and polar interactions. Notably, these include interactions with key residues such as His51 and Ser135, which constitute the catalytic triad.^29^ This finding is relevant, as NS2B-NS3^Pro^ inhibitors have emerged as promising candidates for the development of ZIKV antivirals. In line with this, Yuan et al.^30^ reported that several clinically approved protease inhibitors significantly reduced ZIKV replication. An additional mechanism that deserves consideration is the potential direct interaction of lapachol with viral RNA. Although the present study focused on the binding of lapachol to the NS5 RNA-dependent RNA polymerase, naphthoquinones are known to possess planar aromatic scaffolds capable of interacting with nucleic acids through π-stacking and related noncovalent interactions. Experimental studies have demonstrated that naphthoquinone derivatives can associate with DNA, while the biological activities of this class of compounds have been linked, at least in part, to their ability to modulate nucleic acid-associated processes. Therefore, it is conceivable that lapachol may also interact with viral RNA, either independently or in the context of the NS5 RNA replication complex. Such interactions could potentially alter RNA conformation, accessibility, or polymerase activity, thereby contributing to antiviral effects beyond direct enzyme inhibition. Although this hypothesis was not evaluated in the present work, future studies employing molecular docking, molecular dynamics simulations, and biophysical approaches involving RNA–ligand complexes may help clarify whether RNA binding contributes to the anti-ZIKV activity of lapachol.^31–33^

Regarding the assays addressing early steps of infection, it should be noted that in the virucidal assay, the virus-compound mixture was used to infect cells without a prior dilution step. Given the strong postentry inhibition observed, any carry-over of the compound would be expected to produce some intracellular inhibition; the absence of inhibition in this condition therefore supports a lack of direct virucidal (virion-inactivating) activity of lapachol. A formal dilution step is nonetheless recommended in future experiments to evaluate effects on the viral particle exclusively.^34,35^

In addition, its effect on transcription elongation indicates that lapachol inhibitory properties may be attributed, as well, to direct interference with replication by hindering the activity of NS5^RdRp^. Target-specific molecular docking analysis against this protein predicted interactions with a highly conserved regions, which is essential for enzymatic activity. One of the main relevant findings, with the highest score, is the interactions with Trp805 and Lys802, since they belong to the priming loop region of the NS5^RdRp^, suggesting that lapachol may be hindering its function.^36^ The binding affinity values obtained for lapachol are comparable to those reported for characterized ZIKV RdRp inhibitors in the literature, supporting the feasibility of direct protein–ligand interactions.^37^ Lapachol’s role in viral replication was previously described by our group, where *in silico* evaluations showed its ability to bind to SARS-CoV-2 Nsp9,^38^ a protein whose role involves binding to genomic RNA and preventing its degradation, as well as ZIKV’s NS3 protein.^39^ To date, there are reports of lapachol as an inhibitor of DNA and RNA synthesis, though by interfering with a mitochondrial rate-limiting enzyme in the *de novo* synthesis of pyrimidine ribonucleotides.^40^

Furthermore, our data showed that pretreatment with lapachol protected Vero E6 cells from ZIKV infection, suggesting multiple mechanisms of action at play in addition to the ones described above. One explanation for this protective effect may be attributed to lapachol’s ability to generate ROS, as evidenced by the observed increase in intracellular ROS levels upon treatment. Lapachol can act as an oxidant since its quinone structure, during reduction, forms semiquinone anion radicals, which in turn transfer electrons to molecular oxygen, leading to ROS formation.^17,41^ The ability of lapachol to produce ROS *in vitro* has already been documented.^42,43^ Additionally, the production of ROS as a mechanism of antipathogenic action for lapachol was observed by Linzner and collaborators,^17^ where the compound was able to cause an oxidative stress response, resulting in protein damage in *Staphylococcus aureus*, inhibiting its growth. Furthermore, it must be taken into consideration that oxidative stress may be detrimental to the host.^44^ However, the low levels of ROS observed in uninfected cells combined with the results of the cytotoxicity assays suggest that the compound could be elevating ROS levels in infected cells, enough to act as an inhibitor of viral replication without disrupting the balance in healthy cells. The inhibition of viral replication through ROS production has already been described by Strengert and collaborators,^45^ who demonstrated that ROS production by NADPH oxidases was key in inhibiting Influenza A virus replication by damaging the viral genome. Nonetheless, more studies are needed to further assess the effects of lapachol regarding oxidative stress induction by using *in vivo* models to better evaluate its outcome on the host’s metabolism. Further studies are also needed to directly link ROS formation as a mechanism of action in the inhibition of ZIKV replication.

## Experimental Section

### Lapachol Extraction and Purification

Lapachol was extracted and purified as previously described.^19^ Briefly, Na_2_CO_3_ solution (1% w/v) was added to a mass of 250 g of Purple Ipê (*H. impetiginous*) sawdust. After 24 h undisturbed, the mixture was filtered and HCl (6 M) was added until precipitation occurred. Lapachol was collected by vacuum filtration, recrystallized in ethanol, and exposed to drying for 4 days away from light. The identity and purity of the compound were confirmed by ^1^H and ^13^C NMR spectroscopy, consistent with reported data (Supporting Information).

### Cell Culture and Virus

Vero E6 cells (derived from epithelial kidney cells of African green monkey, ATCC E6) were maintained in Dulbecco’s modified Eagles medium (DMEM, Sigma-Aldrich) supplemented with 100 U/mL of penicillin (Gibco Life Technologies), 100 mg/mL of streptomycin (Gibco Life Technologies), 1% nonessential amino acids (Gibco life technologies), and 5% (v/v) fetal bovine serum (FBS) (Cultilab) in a humidified 5% CO_2_ incubator at 37 °C.

The isolate ZIKV_PE243_, acquired during the 2015 Brazilian outbreak from a patient with classic Zika fever symptoms in Recife, Brazil,^47^ was used for infection assays. Amplification of the virus and determination of viral titers were performed as previously described,^48^ with modifications. Briefly, viral particles were amplified by infecting Vero E6 cells in a 75 cm^2^ flask, incubating for 3 days, and collecting its supernatant. In order to determine viral titers, 10-fold serial dilutions were performed in culture medium and used to infect previously seeded Vero E6 cells in 96-well plates at a density of 5 × 10^3^ cells/well. The infected cells were incubated for 2 h and later overlaid with a culture medium containing 2% carboxymethyl cellulose (CMC) and DMEM 2% FBS for a final 72 h incubation. After this period, focus-forming units per milliliter (FFU/mL) were quantified by an immunofluorescence assay, performed by staining ZIKV NS3 protein, as previously described.^48^

All infection assays were performed at a BSL-2 laboratory under the authorization number CBQ: 163/02 and process SEI: 01.245.006267/2022–14 from the CTNBio—National Technical Commission for Biosecurity from Brazil.

### Cell Viability Assays

To determine cytotoxicity of lapachol, cultured cells at a density of 5 × 10^3^ cells/well on 96-well plates were exposed to concentrations of the compound (diluted in 5% FBS supplemented culture medium) ranging from 0.78 to 100 μM, using a 2-fold serial dilution and incubating at 37 °C for 72 h. After incubation, cell viability was measured using the MTT [3-(4, 5- dimethylthiazol-2-yl)-2, 5-diphenyl tetrazolium bromide] (Sigma-Aldrich) assay. MTT was added at a concentration of 0.5 mg/mL, and after a 30 min incubation at 37 °C, it was replaced with dimethyl sulfoxide (DMSO) to solubilize formazan crystals. Absorbance was measured by optical density (OD) at 560 nm with the Glomax microplate reader (Promega). Cell viability was calculated as (T/C) × 100%, where T and C represent the mean optical density of the treated group and control group, respectively. The cytotoxic concentration of 50% (CC_50_) was calculated by applying nonlinear regression using GraphPad Prism 8.0.^50^

### Antiviral Assay

To assess antiviral activity of lapachol, a dose-dependent assay was performed, as previously described.^48^ Vero E6 cells at a density of 5 × 10^3^ cells/well on 96-well plates were infected with ZIKV_PE243_ at a Multiplicity of Infection (MOI) of 0.01 and simultaneously treated with lapachol at concentrations ranging from 0.78 to 100 μM in 2-fold serial dilutions, followed by incubation at 37 °C. Cells were fixed 72 h postinfection (h.p.i.) using 4% formaldehyde. FFU were determined by immunofluorescence assay.^48^ The effective concentration of 50% (EC_50_) was calculated by applying nonlinear regression using GraphPad Prism 8.0. The values of CC_50_ and EC_50_ were used to calculate the selective index (SI = CC_50_/EC_50_).^50^ A low MOI (0.01) was used in the dose–response antiviral assay so that infection proceeds through multiple replication cycles over the 72 h incubation, allowing the assay to capture the cumulative inhibitory effect of the compound and increasing sensitivity for EC_50_ determination; at higher MOIs, the rapid viral burst overwhelms the monolayer and narrows the window in which compound effects can be distinguished from the control.^51^

### Time-of-Addition Assay

For all time-of-addition protocols, Vero E6 cells were seeded at a density of 5 × 10^3^ cells/well on 96-well plates and incubated overnight at 37 °C, followed by infection with ZIKV_PE243_ in the presence of lapachol at 8 μM, using DMSO 0.1% as untreated control. Upon completion of the 72 h incubation, the supernatant of each replicate was collected and titrated as previously described above.

For pretreatment assessment, cells were exposed to lapachol for 2 h at 37 °C. After this period, cells were washed with phosphate-buffered saline (PBS), to remove any remnants of the compound and infected with the virus at an MOI of 1. The infected cells were then incubated for an additional 2 h, followed by washing with PBS and addition of fresh 2% FBS cell culture medium for a final incubation of 72 h at 37 °C and 5% CO_2_.

For entry/attachment assays, cells were simultaneously infected with ZIKV_PE243_ (MOI = 1) and treated with the compound. After a 2 h incubation, cells were subjected to the same washing and incubation conditions described above. For determination of a virucidal activity, ZIKV_PE243_ at an MOI of 3 was incubated with lapachol at 8 μM for 2 h at 37 °C. Past the incubation period, the virus pre-exposed to the compound was added to the cells, followed by the same protocol outlined for the entry assay. For the postentry assay, seeded cells were infected with the virus at an MOI of 1 and incubated for 2 h, then washed with PBS for unbound virus removal, and incubated with compound-containing medium. Higher MOIs (1 and 3) were used in these time-of-addition assays to ensure sufficient viral inoculum during the short 2 h adsorption windows, guaranteeing reliable infection establishment.^51^ In the virucidal assay, the virus–compound mixture was added directly to the cells without a prior dilution step.

### NS3 Helicase (NS3^Hel^) ATP/GTPase Activity Assay

Assays for the evaluation of ATPase activity of the Helicase domain of NS3 from ZIKV NS3^Hel^ were performed using malachite green; the assay was standardized for NS3^Hel^ as described in the kit manual. Briefly, the ATPase activity of NS3 helicase was measured by quantifying the amount of free phosphate after an NTP hydrolysis. A malachite green assay kit was used as previously described by Cao and co-workers^52^ measuring the absorbance at λ = 620 nm. A reaction mix containing pure DMSO (1% vol/vol) was used as untreated control. In these assays, lapachol was tested at a final concentration of 1 mM, using 200 nM NS3Hel protein and 1 mM ATP as substrate, with a 30 min incubation at 37 °C prior to absorbance reading. Results were analyzed and plotted using GraphPad Prism 8.0.

### NS2B-NS3 Protease (NS2B-NS3^Pro^) Activity Assay

The NS2B-NS3^Pro^ activity assay was performed as previously described by Li and colleagues,^37^ using 96-well black plates. Reactions were carried out in a buffer containing 20 mM Tris (pH 8.5), 10% glycerol, and 0.01% Triton X-100, using 30 μM Benzoyl-Nle-Lys-Arg-Arg-4-methylcoumarin-7-amide (Bz-nKRR-AMC) as the substrate and 4 nM NS2B-NS3^Pro^. During the reaction, the temperature was held at a constant temperature of 37 °C, and fluorescence measurements (excitation λ = 380 nm and emission λ = 460 nm) were performed over a 30 min interval, measuring activity every 60 s.

For initial inhibition assays, lapachol was first diluted in DMSO to a final concentration of 50 mM. For unique-point reaction assays, lapachol was added at a final concentration of 500 μM, resulting in 1% of DMSO in the reaction. Furthermore, to determine the percentage of inhibition by lapachol, protease activity was measured after a period of 20 min and compared to a positive control reaction containing the same amount of DMSO as the reaction containing the ligand. All tests were performed in duplicate. For the concentration–response assay, a similar approach was used; however, 2-fold serial dilutions were performed with concentrations ranging from 0.98 μM to 500 μM, the highest concentration containing 1% of DMSO in the solution. The dose–response curves were analyzed on GraphPad Prism 8.0 using Hill fitting to obtain IC_50_ values.

### ZIKV NS5^RdRp^ Transcription Elongation Activity Assay

ZIKV NS5^RdRp^ activity assay was performed as described by Fernandes and collaborators.^54^ Briefly, transcription elongation activity of NS5^RdRp^ was measured by quantification of SYBR green signal using the synthetic biotinylated self-priming template 3′UTR-U30 (5′-biotin-U30-ACUGGAGAUCGAUCUCCAGU-3′). The end point assays were performed with lapachol at 500 μM. Results were analyzed and plotted using GraphPad Prism 8.0.

### Molecular Docking

Molecular docking was performed with AutoDock Vina v1.1.2 ^55^ to investigate the interactions of lapachol and selected positive controls with the crystal structures of the ZIKV NS3 protease (PDB ID: 5GPI) and NS5 RNA-dependent RNA polymerase (RdRp) (PDB ID: 5U04). Receptor and ligand preparation were carried out using AutoDockTools v1.5.7. For the receptors, water molecules and heteroatoms were removed, followed by the addition of polar hydrogens and assignment of Kollman partial charges. Ligand preparation included the prediction of ionization states at physiological pH (7.4), assignment of Gasteiger charges, merging of nonpolar hydrogens, and definition of torsional degrees of freedom (TORSDOF). For the NS3 protease, the search space was centered on the catalytic residue His51, with coordinates *X* = 70.763, *Y* = 0.199, and *Z* = 46.251. For the NS5^RdRp^, the search space was defined centered near residue Glu804 within the priming loop region, with coordinates *X* = 20.747444, *Y* = 75.664778, and *Z* = 117.665889. For both, grid boxes were defined as a 20 Å × 20 Å × 20 Å dimensions. Docking calculations were performed using an exhaustiveness value of 24, generating up to 40 binding modes (num_modes = 40) within a maximum energy range of 5 kcal/mol. The resulting poses were ranked according to their predicted binding affinities (kcal/mol), and the lowest-energy conformation for each ligand–protein complex was selected for further analysis. Visualization of docking poses and generation of two-dimensional and three-dimensional interaction diagrams were performed using Discovery Studio Visualizer v25.1.0.24284 and PyMOL v3.1.6.1,^56^ respectively.

The docking protocol was validated through redocking of the cocrystallized inhibitors into the binding sites of ZIKV NS3 (PDB: 5LC0) and NS5^RdRp^ (PDB: 6LD4), allowing comparison between the predicted and experimentally determined binding poses (Supporting Information). For the NS5^RdRp^, the redocking of Cpd14 was performed by using an increased exhaustiveness value of 32 to enhance conformational sampling, which reproduced the crystallographic binding orientation of the inhibitor within the allosteric N-pocket.

In addition, the crystallographic inhibitors CN-716 (NS3) and Cpd14 (NS5) were included as positive controls and docked into the corresponding target structures used for lapachol screening under identical docking conditions, including the same grid box dimensions, coordinates, and search parameters (Supporting Information). This approach enabled both the validation of the docking protocol and a direct comparison of the predicted binding modes and interaction profiles of lapachol to those of established inhibitors targeting the same functional regions of each protein.

### Assessment of ROS Production

ROS production was determined using the 2,7′-dichlorohydrofluorescein diacetate (DCFH-DA) fluorescent probe, as previously described with modifications.^57^ Briefly, Vero E6 cells were seeded on a black 96-well plate at a density of 5 × 10^3^ cells/well and left to rest for 24 h. The assay was performed with cells divided into different treatment groups: (a) cells infected with ZIKV_PE243_ at an MOI of 1 in the presence of lapachol at 8 μM; (b) cells infected with ZIKV_PE243_ at an MOI of 1 in the presence of the diluent (DMSO 0.1%); (c) naïve uninfected cells treated with lapachol at 8 μM; (d) naïve uninfected cells treated with the diluent; and (e) untreated and uninfected cells that were used as a blank to establish baseline ROS levels. After a 7 h incubation period at 37 °C, media was removed and cells were washed with PBS, adding 50 μL of the DCHF-DA (10 μM) per well and incubating for 40 min, in the dark, at 37 °C. The probe was then removed, and the cells were washed 5x with PBS 2% Bovine Serum Albumin (BSA), followed by the addition of 50 μL of PBS 1×. Fluorescence at λ_ex_ = 485 nm and λ_em_ = 525 nm was measured in a plate fluorimeter (M2e, Molecular Devices). Results were presented as the ratio of fluorescence intensity at 525 nm normalized to cell density (OD_750_), compared to the untreated control.

### Statistical Analysis

All antiviral experiments in cell culture were performed in triplicate with the repetition of the same conditions a minimum of three times for reproducibility reassurance. For statistical analysis, GraphPad Prism 8.0 was used. Data were analyzed for normal distribution to demonstrate the applicability of the parametric or the nonparametric test. A two-way ANOVA test was employed to compare the treatment of each compound with that of the untreated control (DMSO 0.1%). P value differences of <0.05 were considered significant. EC_50_ and CC_50_ were calculated using a nonlinear regression considering log­(inhibitor) vs response, with a variable slope (four parameters).

## Supplementary Material



## Data Availability

All data supporting the findings of this study are available within the paper and its Supporting Information.

## Notes

^1^H NMR spectrum of lapachol (Figure S1); ^13^C NMR spectrum of lapachol (Figure S2); FTIR spectrum of lapachol (Figure S3); predicted binding modes and interaction networks of the reference inhibitors CN-716 and Cpd14 against ZIKV NS2B-NS3^Pro^ and NS5^RdRp^ (Figure S4); redocking of the cocrystallized ligand into the ZIKV NS3 binding site, comparing experimental and predicted poses (Figure S5); redocking of the cocrystallized ligand into the ZIKV NS5^RdRp^ binding site, comparing experimental and predicted poses (Figure S6); estimated binding affinity scores and interaction profiles for all poses generated in the molecular docking simulations of lapachol against ZIKV NS2B-NS3^Pro^ and NS5^RdRp^ (Table S1) (PDF)

## #

In memoriam of Diego Pandeló José.
