# Supplementary material for: Insights into Inhibitory Effects of Lapachol against Zika Virus
Source: ACS Omega. 2026 Oct 5;11(41):62804–14. doi: 10.1021/acsomega.6c04563 (PMC13689105; doi:10.1021/acsomega.6c04563)
Supplement: Supplementary file 1 [file ao6c04563_si_001.pdf]

INSIGHTS INTO INHIBITORY EFFECTS OF LAPACHOL AGAINST ZIKA VIRUS

Stephanie Paola Borjas Reyes[1], Natasha Marques Cassani[1], João Victor Rodrigues Dutra[2], Maria Eduarda Trevisan Aguillar da Silva[3], Nathalya Cristina de Moraes Roso Mesquita[4], Caroline Martins Mota[5], Tiago Wilson Patriarca Mineo[5], Glaucius Oliva[4], Rafael V. C. Guido[4], Bruno Amaral Meireles[3], Diego Pandeló José[3†], Ana Carolina Gomes Jardim[1*].

[1] Laboratory of Antiviral Research (LAPAV), Institute of Biomedical Science (ICBIM), Federal University of Uberlândia (UFU), Uberlândia, MG, Brazil.

[2] Federal University of Minas Gerais, Institute of Biological Sciences, Department of Genetics, Ecology and Evolution, Belo Horizonte, Minas Gerais, Brazil.

[3] Federal University of Triângulo Mineiro, Iturama, Minas Gerais, Brazil.

[4] Laboratory of Structural Biology and Drug Discovery, Center for Research and Innovation in Biodiversity and Drugs (CIBFar/CEPID), Institute of Physics of São Carlos, University of São Paulo, São Paulo, Brazil.

[5] Laboratory of Immunoparasitology "Dr. Mário Endsfeldz", Federal University of Uberlândia (UFU), Uberlândia, MG, Brazil.

***Corresponding author:** Professor Ana Carolina Gomes Jardim, PhD. Laboratory of Antiviral Research, ICBIM, UFU. Address: Amazonas Avenue, 4C building – room 216, Umuarama, Uberlândia, MG, Brazil, CEP: 38405-302. Tel.: +55 (34) 3225-8682. E-mail: jardim@ufu.br

Table of Contents

Figure S1. ^{1}H NMR spectrum of lapachol (400 MHz, $CDCl_3$)

Figure S2. ^{13}C NMR spectrum of lapachol (100 MHz, $CDCl_3$)

Figure S3. FTIR spectrum of lapachol

Figure S4. Predicted binding modes of the reference inhibitors CN-716 and Cpd14 with ZIKV NS3 protease and NS5 RNA-dependent RNA polymerase

Figure S5. Validation of the molecular docking protocol for the ZIKV NS2B-NS3 protease

Figure S6. Validation of the molecular docking protocol for the ZIKV NS5 RNA-dependent RNA polymerase (RdRp)

Table S1. Binding affinities for ZIKV NS3 and NS5

Supplementary material

^{1}H and ^{13}C NMR spectroscopy

A 1% (w/v) aqueous solution of sodium carbonate (Na_2CO_3) was added to 250 g of *Handroanthus impertiginosus* (Ipê Roxo) powder and allowed to stand for 24 h. After this period, the solution was filtered and acidified with 6 M hydrochloric acid until precipitation of a yellow solid. The crude lapachol was collected by vacuum filtration and recrystallized from hot ethanol. The resulting solid was dried under light protection for 4 days to afford 4.5 g of a yellow compound (1.8% yield) as the sole isolated substance.

1H NMR (400 MHz, CHLOROFORM-d) δ ppm 1.66 - 1.71 (m, 4 H) 1.79 (s, 3 H) 3.31 (d,J=7.35 Hz, 3 H) 5.16 - 5.26 (m, 1 H) 7.36 (s, 1 H) 7.62 - 7.70 (m, 1 H) 7.74 (td, J=7.55, 1.39 Hz, 1 H) 8.06 (dd, J=7.55, 0.99 Hz, 1 H) 8.11 (dd, J=7.75, 0.79 Hz, 1 H)

13C NMR (100 MHz, CHLOROFORM-d) δ ppm : 17,84; 22,59; 25,70; 119,63; 123,46; 126,00; 126,72; 129,42; 132,79; 134,78; 152,67; 181,65; 184,48

This report was created by ACD/NMR Processor Academic Edition. For more information go to www.acdlabs.com/nmrproc/

**Formula** $C_{15}H_{14}O_3$	**FW** 242.2699

**Acquisition Time (sec)**	5.0332	**Comment**	BMPP 001 (2024-07-08) CDCL3		
**Date**	08 Jul 2024 11:51:44			**Date Stamp**	08 Jul 2024 11:51:44
**File Name**	C:\Users\Bruno\Desktop\RMN\BMPP 001 (2024-07-08)\1\fid				
**Frequency (MHz)**	400.13	**Nucleus**	1H	**Number of Transients**	64
**Origin**	spect	**Original Points Count**	32768	**Owner**	root
**Points Count**	32768	**Pulse Sequence**	zg	**Receiver Gain**	3.93
**SW(cyclical) (Hz)**	6510.42	**Solvent**	CHLOROFORM-d		
**Spectrum Offset (Hz)**	2824.5647	**Spectrum Type**	STANDARD	**Sweep Width (Hz)**	6510.22
**Temperature (degree C)**	30.000				

^{1}H NMR (400 MHz, CHLOROFORM-*d*) δ ppm 1.71 (br. s., 0 H) 1.66 - 1.71 (m, 4 H) 1.79 (s, 3 H) 3.31 (d, *J*=7.35 Hz, 3 H) 5.16 - 5.26 (m, 1 H) 7.36 (s, 1 H) 7.62 - 7.70 (m, 1 H) 7.74 (td, *J*=7.55, 1.39 Hz, 1 H) 8.06 (dd, *J*=7.55, 0.99 Hz, 1 H) 8.11 (dd, *J*=7.75, 0.79 Hz, 1 H)

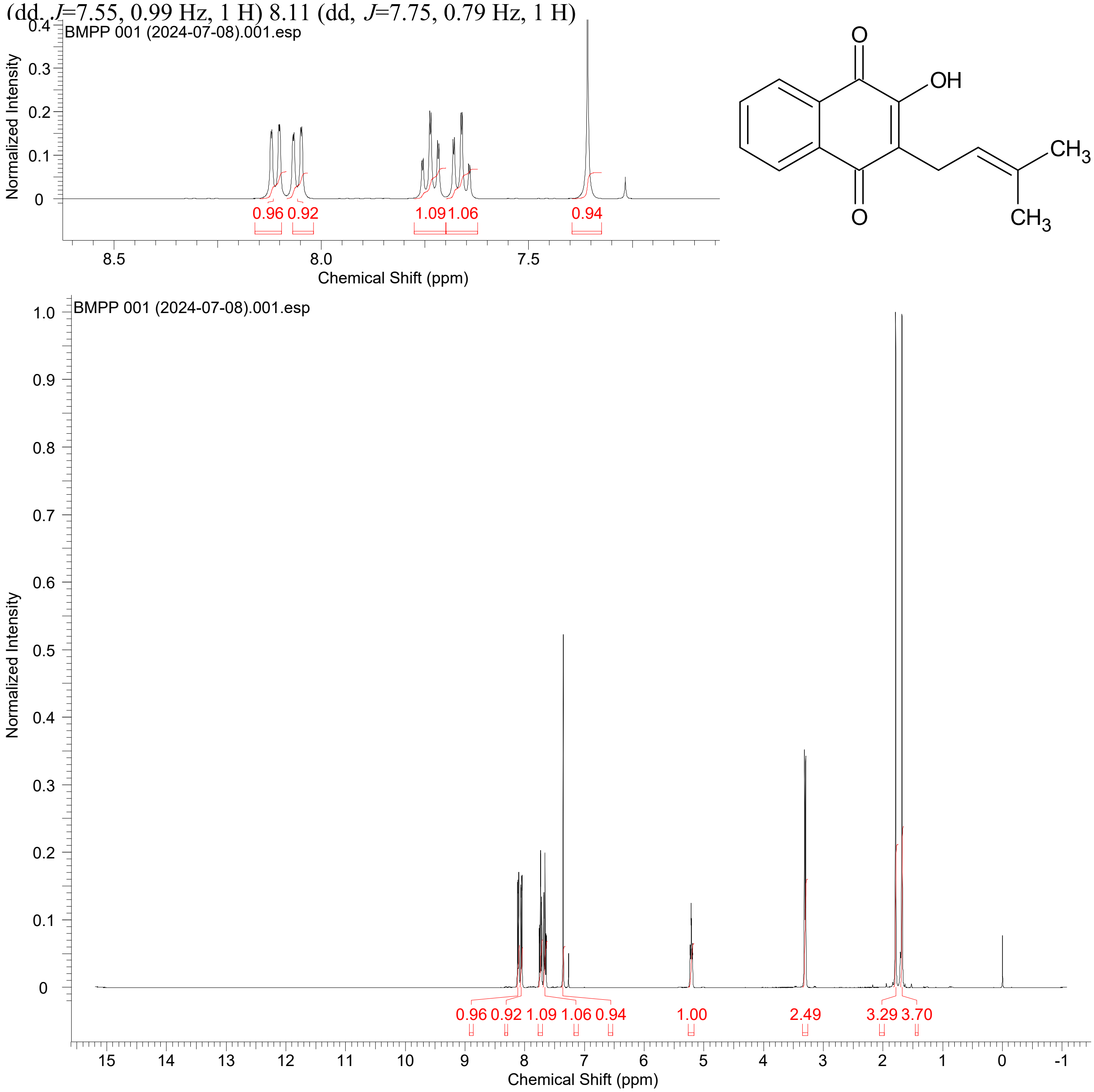

Figure S1. ^{1}H NMR spectrum of lapachol (400 MHz, $CDCl_3$). The lower panel shows the full spectrum and the upper panel an expansion of the aromatic region (7.2–8.6 ppm), with the corresponding integration values. The chemical structure of lapachol is shown for reference.

This report was created by ACD/NMR Processor Academic Edition. For more information go to www.acdlabs.com/nmrproc/

Formula	$C_{15}H_{14}O_{3}$	***FW***	242.2699

Acquisition Time (sec)	0.6488	***Comment***	BMPP 001 (2024-07-08) CDCL3			***Date***	08 Jul 2024 13:46:56
Date Stamp	08 Jul 2024 13:46:56			***File Name***	C:\Users\Bruno\Desktop\RMN\BMPP 001 (2024-07-08)\2\fid		
Frequency (MHz)	100.62	***Nucleus***	13C	***Number of Transients***	8192	***Origin***	spect
Original Points Count	16384	***Owner***	root	***Points Count***	16384	***Pulse Sequence***	zgpg30
Receiver Gain	198.02	***SW(cyclical) (Hz)***	25252.52	***Solvent***	CHLOROFORM-d		
Spectrum Offset (Hz)	11064.1250	***Spectrum Type***	STANDARD	***Sweep Width (Hz)***	25250.98	***Temperature (degree C)***	30.002

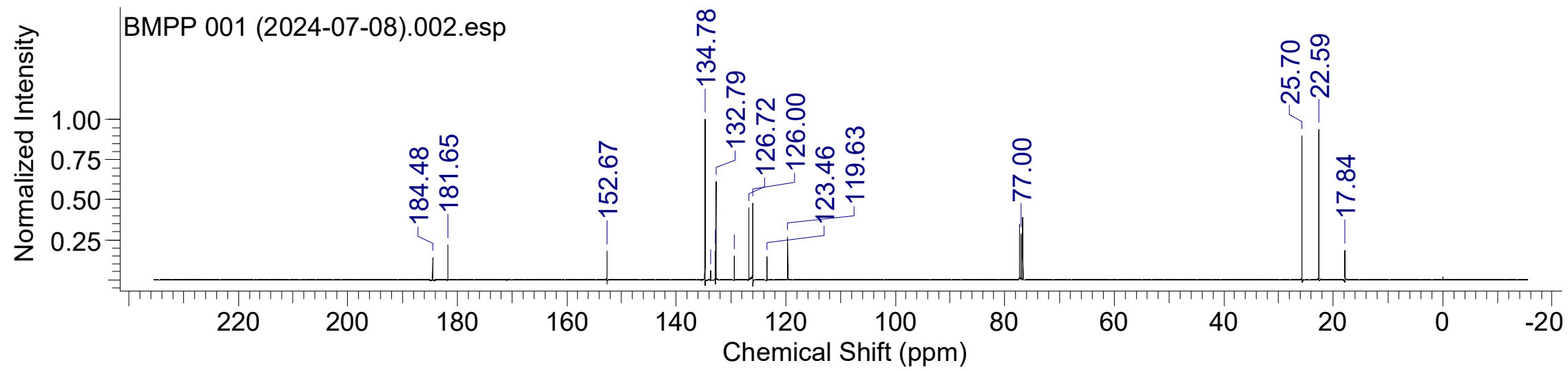

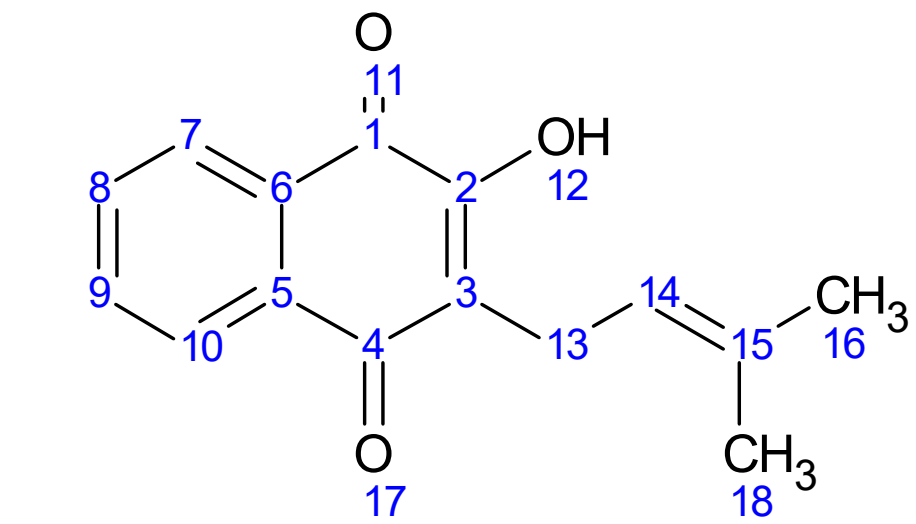

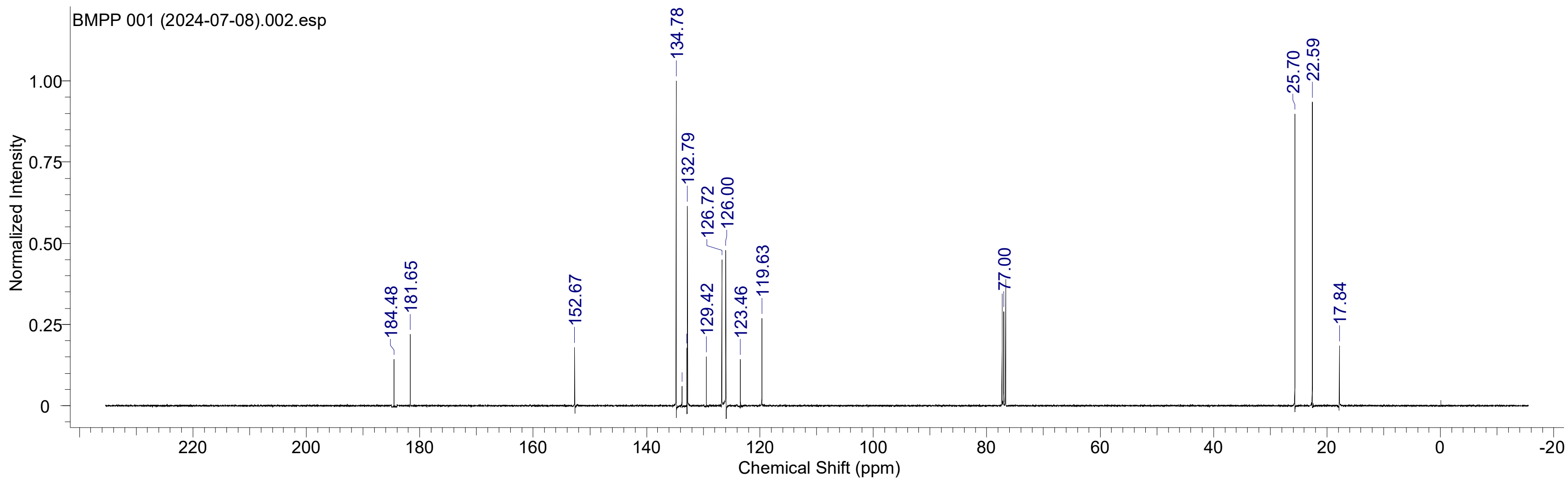

Figure S2. ^{13}C NMR spectrum of lapachol (100 MHz, $CDCl_3$). The lower panel shows the full spectrum and the upper panel the same spectrum with the chemical shift values labelled. The numbered structure indicates the carbon numbering used for the assignments. The signal at 77.00 ppm corresponds to the $CDCl_3$ solvent.

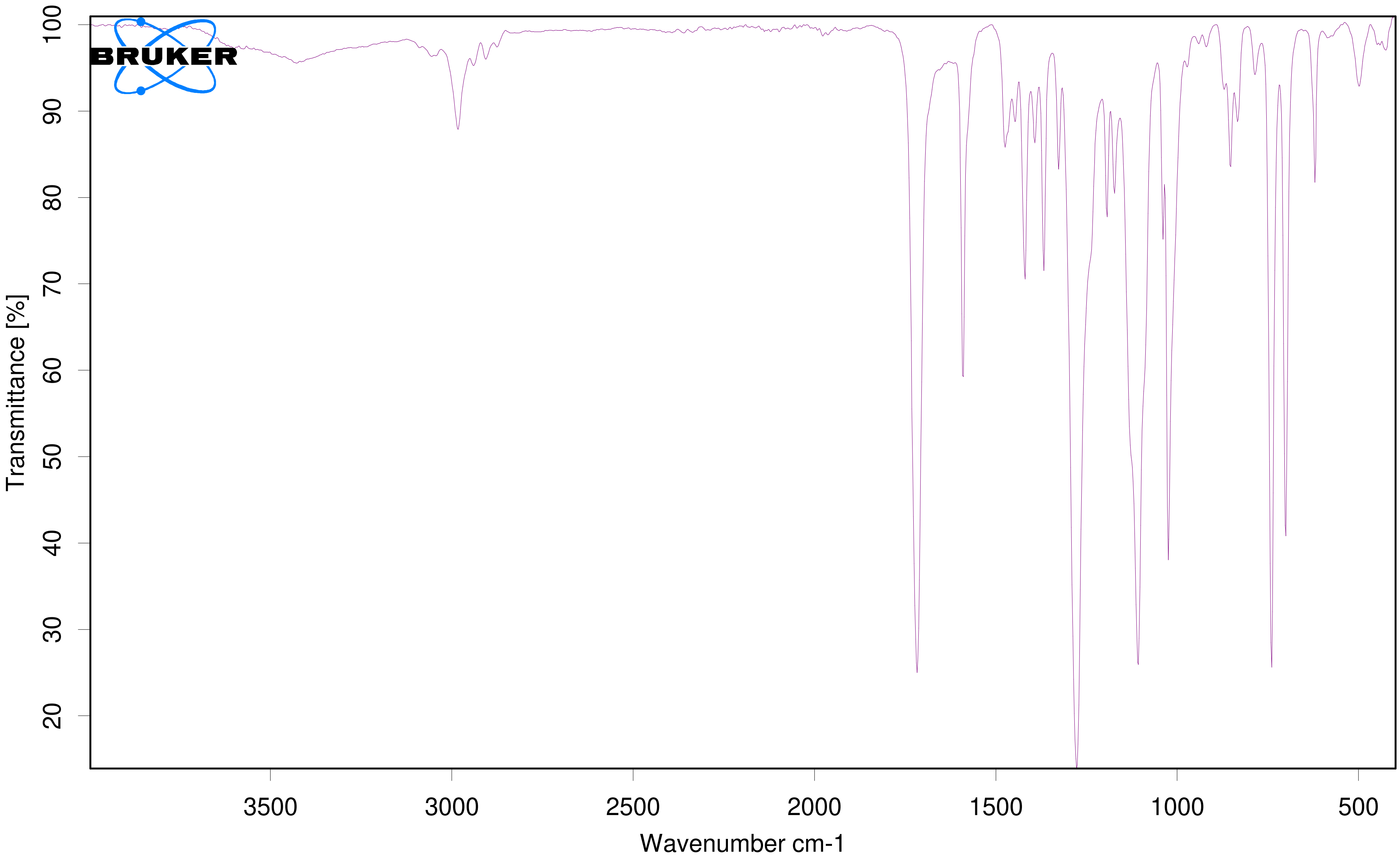

C:\Users\ADMUFTM\Documents\Espectros FTIR\Prof Pedro\Alunos\Lorraine\BMPP000.0 BMPP000 Lorraine 15/03/2024

Page 1 of 1

Figure S3. FTIR spectrum of lapachol, presented as transmittance (%) as a function of wavenumber (cm^{-1}) over the 4000–400 cm^{-1} range.

Binding modes of reference inhibitors within ZIKV targets

To validate the docking protocol and provide a structural reference for comparison with lapachol, molecular docking was performed using the known ZIKV inhibitors CN-716 and Cpd14 against the NS3 protease and NS5 RNA-dependent RNA polymerase (RdRp), respectively (**Figure S4A**). CN-716 was predicted to bind within the catalytic pocket of the NS3 protease domain, occupying a cavity adjacent to the catalytic residue His51. The ligand established conventional hydrogen bonds with Arg59 and Ser56, while additional stabilization was provided by carbon hydrogen bonding with Thr53, Ala57, and Leu58. Multiple van der Waals contacts involving residues Tyr23, Arg24, Ser60, Val36, Val52, and Phe46 further contributed to ligand accommodation within the binding pocket. Hydrophobic interactions, including π-alkyl contacts with Val25 and Arg28, and π-sigma and alkyl contacts with Leu58 were also observed. An unfavorable donor–donor interaction involving Arg59 was identified, suggesting a minor steric or electrostatic constraint within the binding pose. Overall, the predicted binding mode is consistent with the occupation of the enzymatically relevant region of the NS3 protease.

For the NS5 RdRp, Cpd14 was predicted to bind within a pocket located adjacent to the priming loop, a structural element essential for de novo RNA synthesis initiation (**Figure S4B**). The ligand formed conventional hydrogen bonds with Thr795 and Thr796 and established additional van der Waals contacts with Gly793 and Thr792, while additional stabilization was provided by carbon hydrogen bonding with Asn407 and Gly801 were observed. Hydrophobic stabilization was mediated by π-alkyl and π-sigma interactions with Arg794 and Met806, respectively, while a π-sulfur interaction with Met806 further reinforced ligand binding. An unfavorable donor–donor interaction involving Trp805 was also detected. The docking pose positioned Cpd14 deeply within the priming loop-associated cavity, in agreement with previous reports describing this compound as an allosteric inhibitor of *Orthoflavivirus* polymerases. Collectively, these results demonstrate that both reference compounds occupy functionally relevant regions of their

respective targets, supporting the reliability of the docking protocol employed in this study.

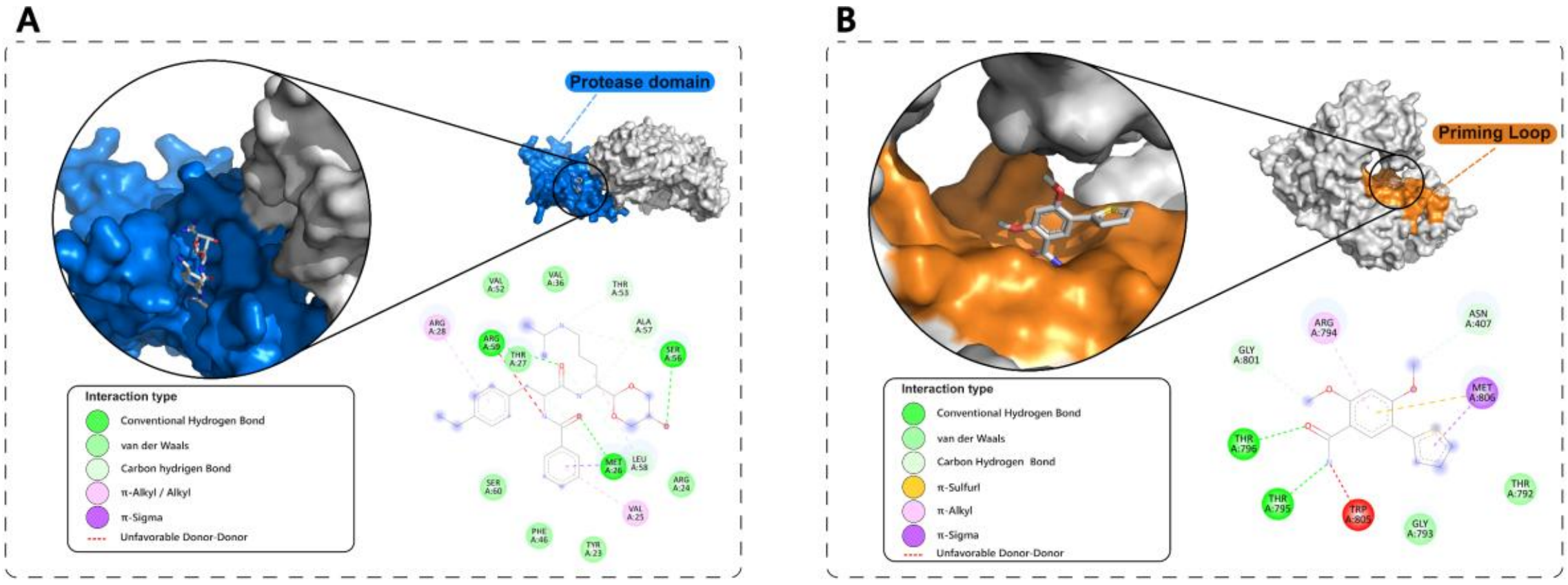

Figure S4. Predicted binding modes of the reference inhibitors CN-716 and Cpd14 with ZIKV NS3 protease and NS5 RNA-dependent RNA polymerase. (**A**) Three-dimensional representation of the docking pose of CN-716 within the protease domain of the Zika virus (ZIKV) NS3 protein. The protease domain is highlighted in blue, while the remaining protein regions are shown in gray. The enlarged view illustrates the ligand accommodated within the catalytic pocket in proximity to the enzymatically active region. The corresponding two-dimensional interaction diagram depicts the network of conventional hydrogen bonds, carbon hydrogen bonds, van der Waals contacts, π-alkyl, and π-sigma interactions contributing to ligand stabilization. An unfavorable donor–donor interaction is also indicated. (**B**) Predicted binding mode of Cpd14 within the RNA-dependent RNA polymerase (RdRp) domain of ZIKV NS5. The priming loop region is highlighted in orange on the protein surface, and the enlarged view shows the ligand occupying a cavity adjacent to this functional element. The two-dimensional interaction map reveals conventional hydrogen bonds with Thr795 and Thr796, together with van der Waals, π-alkyl, π-sigma, and π-sulfur interactions involving residues surrounding the priming loop region, including Arg794, Met806, Gly793, Gly801, Asn407, and Thr792. An unfavorable donor–donor interaction with Trp805 is also observed. Interaction types are color-coded according to the legends shown in each panel. These docking results reproduce the binding of previously reported inhibitors within catalytically and functionally relevant regions of NS3 and NS5, supporting the suitability of the molecular docking strategy used in this study.

The docking protocol was additionally validated by redocking the co-crystallized inhibitors into the binding sites of ZIKV NS2B-NS3 (PDB: 5LC0) (**Figure S5**) and NS5 RdRp (PDB: 6LD4) (**Figure S6**). The simulations successfully reproduced the experimental binding modes with RMSD values of 1.74 Å and 2.374 Å, respectively.

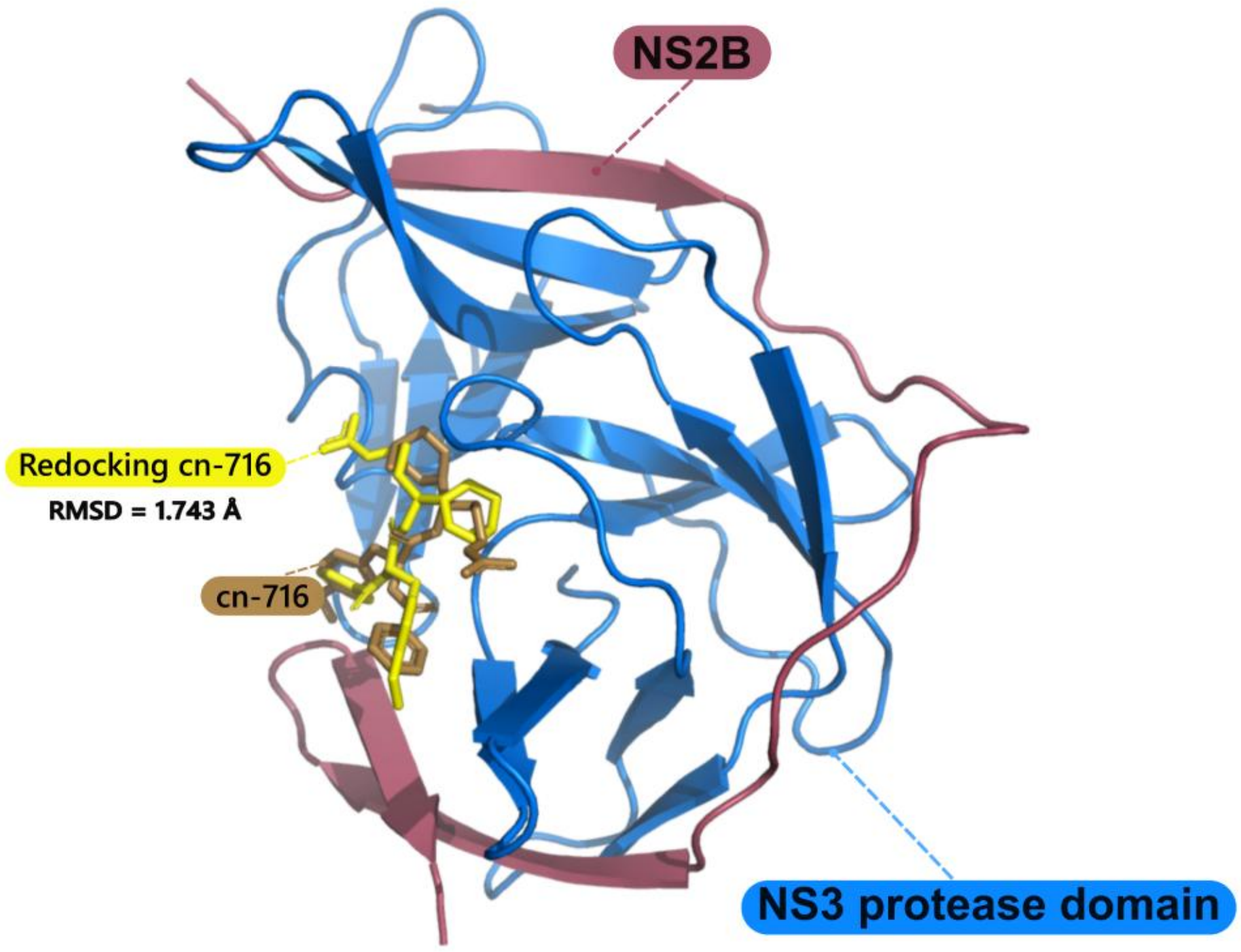

Figure S5. Validation of the molecular docking protocol for the ZIKV NS2B-NS3 protease. Superimposition of the crystallographic pose (brown) and the predicted redocking pose (yellow) of the potent boronate inhibitor cn-716 (PDB ligand ID: 6T8) within the catalytic cleft of the ZIKV NS2B-NS3 protease (PDB: 5LC0). The AutoDock Vina search parameters successfully reproduced the native binding mode, yielding a highly accurate Root Mean Square Deviation (RMSD) of 1.743 Å for the heavy atoms. The NS3 protease domain is highlighted in blue, and the essential NS2B cofactor is shown in dark red.

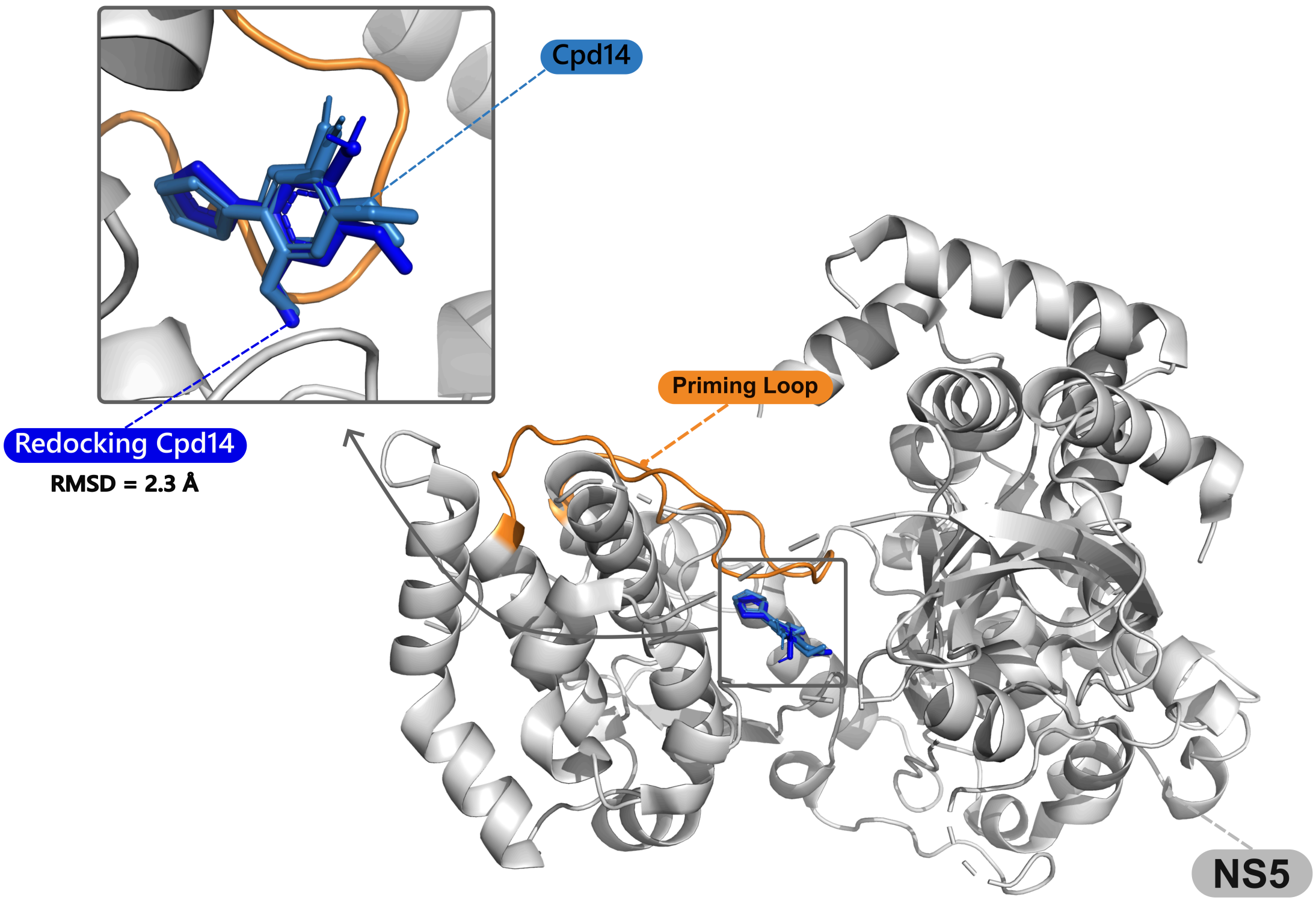

Figure S6. Validation of the molecular docking protocol for the ZIKV NS5 RNA-dependent RNA polymerase (RdRp). Superimposition of the native crystallographic pose (dark blue) and the predicted redocking pose (light blue) of the non-nucleoside inhibitor Compound 14 (Cpd14; PDB ligand ID: G8O) within the allosteric N-pocket of the ZIKV NS5 RdRp (PDB: 6LD4). The docking simulation yielded an RMSD of 2.374 Å. This slight structural deviation is thermodynamically consistent with the highly solvated nature of the native N-pocket, which utilizes a network of non-labile water molecules to anchor the inhibitor. In the simulated desolvated environment, the flexible ligand exhibits a minor conformational tilt to compensate for the absent structural waters, accurately confirming the spatial targeting of the N-pocket adjacent to the priming loop (orange).

Table S1: Binding affinities for ZIKV NS3 and NS5

NS3				NS5			
**Pose**	**Affinity (kmol/mol)**	**Dist. fromRMSD l.b.**	**Dist. fromRMSD u.b.**	**Pose**	**Affinity (kmol/mol)**	**Dist. fromRMSD l.b.**	**Dist. fromRMSD u.b.**
1	-6.232	0	0	1	-5.998	0	0
2	-6.202	0.995	1.327	2	-5.908	1.176	2.922
3	-6.162	1.231	1.617	3	-5.875	1.481	2.228
4	-6.159	1.14	1.811	4	-5.704	1.325	6.294
5	-5.848	1.919	2.706	5	-5.69	10.94	12.46
6	-5.592	1.262	3.075	6	-5.595	3.09	7.502
7	-5.587	3.225	4.521	7	-5.565	14.39	15.54
8	-5.344	3.759	5.208	8	-5.549	11.32	13.67
9	-5.311	3.637	6.397	9	-5.539	11.4	13.69
10	-5.118	4.631	7.057	10	-5.526	1.373	5.476
11	-5.077	2.022	6.048	11	-5.516	13.87	14.67
12	-5.013	3.382	4.271	12	-5.455	1.913	5.667
13	-4.986	1.891	2.545	13	-5.441	1.137	2.841
14	-4.963	4.104	6.313	14	-5.425	12.81	15.22
15	-4.946	1.892	6.701	15	-5.404	11.39	13.5
16	-4.933	2.732	4.137	16	-5.33	1.516	5.677
17	-4.916	1.64	3.218	17	-5.325	3.735	5.943
18	-4.878	2.889	3.495	18	-5.308	11.09	12.86
19	-4.803	4.415	6.044	19	-5.268	11.52	12.77
20	-4.775	2.965	3.754	20	-5.25	11.82	13.19
21	-4.743	4.867	6.876	21	-5.241	11.56	12.95
22	-4.698	2.162	6.482	22	-5.215	11.65	14
23	-4.663	4.586	6.956	23	-5.201	12.12	13.24
24	-4.647	5.611	6.691	24	-5.175	13	15.26
25	-4.636	1.725	6.593	25	-5.16	12.06	14.29
26	-4.612	4.113	5.69	26	-5.099	12.24	13.97
27	-4.611	10.19	14.65	27	-5.095	11.89	14.53
28	-4.609	2.093	2.94	28	-5.067	4.532	7.156
29	-4.595	2.143	3.103	29	-5.024	12.31	14.3
30	-4.571	5.569	8.332	30	-4.994	10.45	12.81

31	-4.57	3.546	5.268
32	-4.57	3.403	5.949
33	-4.531	2.65	6.265
34	-4.529	5.406	6.535
35	-4.516	2.768	3.829
36	-4.508	2.243	6.126
37	-4.479	4.081	5.149
38	-4.479	3.007	3.913
39	-4.458	2.216	5.832
40	-4.368	2.862	4.252

31	-4.975	10.77	11.94
32	-4.943	6.476	10.02
33	-4.896	11.84	13.72
34	-4.882	11.51	14.04
35	-4.87	12.58	14.19
36	-4.827	2.017	2.606
37	-4.82	12.47	14.03
38	-4.81	12.12	14.13
39	-4.767	13.34	15.65
40	-4.73	2.853	4.131
